# Climate and Clothing

**Published:** 1900-04-28

**Authors:** 


### The Hospital

A Journal of

The Medical Sciences and Hospital Administration.

Vol. XXVIII.—No. 709.] Edited by Sir Henry Burdett, K.C.B., and Solomon C. Smith, M.D., M.R.C.P. [April 28, 1900.

### Climate and Clothing.

In those years of which the present appears to be an example, in which

"Upon the first warm day,
Which sunny April in its train doth bring,"

the thermometer suddenly ascends from among the fifties to among the seventies, and then as suddenly runs down again, there is probably no matter of more impo[illegible]ance to health than that proper attention should [illegible] paid to the adoption of clothing suitable to the char[illegible]ing conditions of the season. A century ago, among th[illegible]e who desired to be considered good housewives, the use of domestic fires began and ceased in more dependence upon the calendar than upon the weather. They were not lighted in sitting-rooms until November 1st, nor were they continued after April 1st. In like manner, the commencement of summer clothing was determined by certain feasts of the Church, especially by Easter Day and Whit Sunday, rather than by anything so trivial as considerations of external temperature. In both respects usage has undergone many changes for the better, and the sitting-room fire is now a chartered libertine, liable to be kindled whenever the air is cold, and to be suffered to expire unnoticed when it is genial. In respect of clothing there has been some progress, but not to an equivalent degree; and this mainly because it is so largely controlled by the mysterious goddess slavishly worshipped by the fair sex under the name of fashion, who so often demands and receives from her votaries tributes of the same kind which are paid to other idols. Children are sacrificed to fashion as ruthlessly as they once were to Moloch; and adults as ruthlessly as they once were to Juggernaut. A sudden occurrence of heat in early April will send scores of ladies out to take exercise in hot and heavy garments, for no better reason than that the only cool ones which they possess were made "last season," and will betray, to the keen appreciation of feminine eyes, that they are not of the mode which is expected to prevail in the coming May. Even with those who are more prudent, the resurrection of summer garments may often mean the peaceful burial of winter ones; and then, when after a spell of hot weather there is a return of actual or comparative cold before the summer fairly establishes its reign, we have at least considerable risk of inadequacy of protection. In our uncertain climate, with its sudden and capricious changes, it is probable that a very large portion of the illnesses of the community, and especially of the illnesses affecting the respiratory tract, are in great measure due to want of harmony between the state of the temperature and the warmth and thickness of the dress.

The many people who, during the hot weather of last week, took their accustomed exercise in the clothing which they had worn in March would assuredly agree in their complaints of the lassitude and disinclination for effort which were produced; and a large proportion of them, to whom this exercise would be rendered compulsory by considerations arising from business or employment, would be quite certain to suffer in an appreciable degree from the subsequent chilling of an overheated body. "Insufficient, improper, or excessive clothing," wrote the authors of a very admirable article in "Quain's Dictionary," "are most important sources of predisposition to disease, amongst the rich as well as amongst the poor;" for, although the climate is so variable, corresponding adaptations of dress are for the most part neglected.

We have often thought that the best possible evidence of care and skill on the part of a family doctor is the rare occurrence of cases of serious illness among the patients who are really subject to the influence of his advice; and there are few departments of domestic management as to which advice is more needed than with reference to the management of dress. The common sense, on which not a few mothers would be prepared to plume themselves, and from which they would expect to derive sufficient enlightenment for the guidance of conduct, either exercises but little sway over the wardrobe, or its dictates are habitually neglected. It affords no protection against the wiles of advertisers, who, armed in complete panoply of sham science, strain every nerve to force their various fabrics upon the notice and acceptance of the public. Still less does it afford protection against the modern forms of Moloch already referred to, even when the priests of these deities require that the knee-joints of little children should be unclothed, and left exposed to every change and inclemency of weather. Something, of course, must always be allowed for individual peculiarities; and one member of a family may often require more protection than another. But, as a general principle, it cannot be wrong, and would certainly often be safe, to regard a thermometer as a normal part of the furniture of a dressing-room; and to see that the warmth and the weight of clothing bore at least some definite relation to its indications.

